# Early Experience of Reverse Total Shoulder Arthroplasty in a Public Hospital in Malaysia

**DOI:** 10.5704/MOJ.2103.018

**Published:** 2021-03

**Authors:** CX Sam, AZ Anwar, AR Ahmad, GN Solayar

**Affiliations:** 1 Department of Orthopaedics, International Medical University, Seremban, Malaysia; 2 Department of Orthopaedics, Tuanku Ja'afar Hospital, Seremban, Malaysia

**Keywords:** replacement, proximal humerus, rotator cuff, arthropathy, shoulder fractures

## Abstract

**Introduction:**

Reverse total shoulder arthroplasty provides a surgical alternative to standard total shoulder arthroplasty for the treatment of cuff tear arthropathy, arthritis and fracture sequelae. This study aimed to assess the short-term outcomes following reverse total shoulder arthroplasty for patients in a large public hospital in Malaysia.

**Materials and Methods:**

We identified and performed five primary reverse total shoulder arthroplasties between 1 May 2019 and 1 June 2020. All patients were contactable and available for analysis. Assessment of functional outcomes was performed using the Constant-Murley score, the patient satisfaction score (PSS), and imaging studies. The mean follow-up from operation to the time of reporting was 9.6 months (range, 3 to 14 months)

**Results:**

The median age for our patients was 58 years (±11.91). The most common indication for surgery was post-traumatic arthritis, followed by rotator cuff arthropathy and osteoarthritis. The mean Constant score improved from 9.0 pre-operatively to 52.3 post-operatively at a mean of 9.6 months. The majority of the patients were satisfied with the surgery as the post-operative range of motion, especially anterior elevation and abduction, improved in four of our patients and there were no short-term complications, for example, of infection or revisions, reported at the last follow-up.

**Conclusion:**

This study has shown that reverse total shoulder arthroplasty can yield good short-term outcomes for the treatment of complex shoulder problems in addition to cuff tear arthropathy. It should be considered a treatment for rotator cuff tears, severe arthritis and ≥ 3 parts proximal humeral fractures.

## Introduction

Shoulder arthroplasty is a well-established procedure for patients with degenerative glenohumeral arthritis or traumatic fractures^1,2^. In most cases, shoulder arthroplasty provides excellent pain relief and improved functional outcomes. Generally, shoulder arthroplasties can be divided into the anatomic (total or hemi-) arthroplasty or nonanatomic (reverse) arthroplasty. Anatomic shoulder arthroplasty in the presence of complete and irreparable rotator cuff tears frequently yields unsatisfying results. Reverse total shoulder arthroplasty (RTSA) has emerged as an alternative option for end-glenohumeral shoulder pathology, with increasing application in the developed countries like Australia, New Zealand, Finland and the United States^1-5^. Indications for RTSA include arthropathy, grossly deficient rotator cuff, osteoarthritis, rheumatoid arthritis and post-traumatic arthritis of the shoulder^4,6,7^.

Reverse total shoulder arthroplasty (RTSA) has demonstrated promising short and medium-term functional outcomes^8-13^. However, the long-term results of RTSA have rarely been reported. Although some studies have shown acceptable long-term prosthetic survivorship after RTSA, concerns regarding clinical and radiological complications, such as notching and loosening, have been raised, especially in high demand patients. Consequently, RTSA is frequently performed in patients who are elderly with low demand and have rotator cuff tear arthropathy^8,9,14^.

From a database search in PubMed, Scopus, and Web of Science, there are no studies on the outcomes of RTSA published in Malaysia to our knowledge. With rapidly changing patient demographics, the number of complex glenohumeral shoulder cases is expected to rise. The purpose of this study was to evaluate the clinical and radiological outcomes of RTSA in a tertiary hospital in Malaysia.

## Material and Method

This retrospective study was conducted in Hospital Tuanku Jaafar (HTJ) Seremban, Negeri Sembilan, Malaysia. A total of five patients with rotator cuff arthropathy, osteoarthritis, and fracture sequelae was identified from 1 May 2019 to 1 June 2020. All five patients were contacted and analysed. All patients were followed up for a mean of 9.6 months. Our institutional ethics committee approved this study, and all patients provided written informed consent to share their data in the study. Before surgery, the options of non-operative management were discussed with all patients. The inclusion criteria were patients aged 18 years and older, patients with a grossly deficient rotator cuff tear, severe arthritis and ≥ 3 parts proximal humeral fractures. This study excluded all absolute contraindications for revision total shoulder replacement such as infections, sepsis and osteomyelitis. Other exclusion criteria included patients with evidence of tumours, uncooperative patients or patients with neurologic disorders who were incapable of following verbal instructions.

All surgeries were done by a single fellowship-trained surgeon. (GNS) All patients received the Comprehensive® Reverse Shoulder System [Zimmer Biomet, USA]. The deltopectoral approach was used in all five shoulders. Associated procedures were biceps tenotomy and subscapularis trans-osseous repair using two Ethibond suture at the time of closure. All humeral stems were of standard length (100mm); all were nine mm in diameter. An All-Glenoid base plate with an uncemented central canal was used. The glenoid base plate was fixed to the glenoid via three screws. Post-operative care was standardised. Passive range of motion was started one day post-operatively. Active flexion and abduction beyond 90° were allowed after six weeks.

All patients were evaluated by the surgeon and sports physicians for a mean of six months following surgery. All five patients were asked to fill in overall subjective outcomes via a self-administered questionnaire after surgery with the assistance of a medical officer. The patient's response was collected and finalised through a telephone interview. Patients rated their overall subjective outcomes as “excellent,” “good,” “fair,” or “unsatisfied”.

Clinical assessment included assessment for a range of motion, strength of the shoulder and functional activity. These were quantified using the Constant-Murley scoring system. Radiological evaluation included anteroposterior views of the glenohumeral joint in the neutral position. Outcomes evaluated from post-operative radiographs were for the presence of fractures, loosening, dislocations and notching.

data were recorded as mean (±standard deviation, SD) or as median followed by interquartile range as data were not normally distributed. The Wilcoxon signed ranked test was used as appropriate to analyse the variance of the difference between pre-reverse total shoulder arthroplasty (RTSA) and post RTSA. A p-value of less than 0.05 was considered to be statistically significant.

## Results

**Fig. 1:**
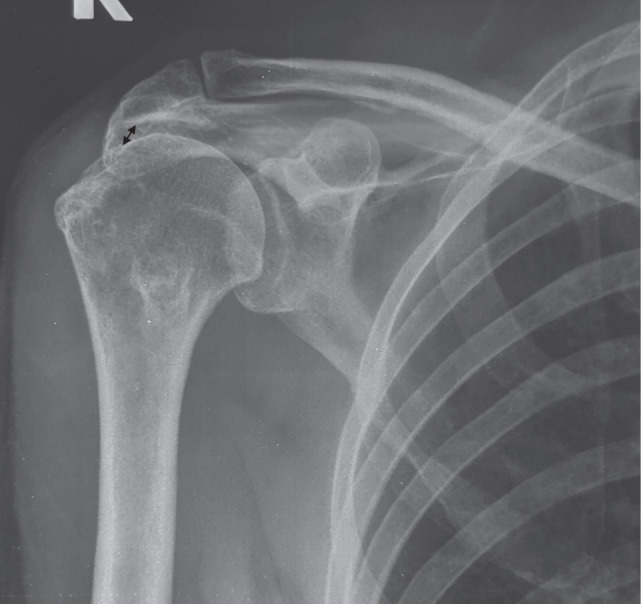
Anterior-posterior view of a shoulder radiograph showing narrowed acromio-humeral interval (arrow), which is superior migration of the humeral head indicating rotator cuff arthropathy. Intra-operatively, the rotator cuff was found irreparably destroyed.

Three of the five patients were male, and the median age of our patients was 58.0±11.19 years. All five patients were right-handed, and all had the dominant side involved. Four patients suffered from ≥ 3 parts proximal humeral fractures treated conservatively, while two had rotator cuff arthropathy, and one of them with osteoarthritis (Fig. 1). Their duration from injury to surgery ranged from six months to two years. The mean duration of follow-up was 9.6 months (range 3 to 14). There were no short-term surgical complications reported in this cohort. Four out of the five patients were satisfied with the outcomes of their shoulder surgery.

The Constant-Murley scores improved from nine (range 3 to 15) points pre-operatively to 53.2 (range 16 to 78) post-operatively, and this was statistically significant Table I. Shoulder pain was significantly reduced in all patients, allowing undisturbed sleep post-operatively.

**Table I:** Pre-operative and post-operative functional shoulder assessment

Constant score	Patient number					Mean(range)
	1	2	3	4	5	
Pain						
Pre-op	0	3	1	3	0	1.4 (0 to 3)
Post-op	6	11	5	6	0	5.6 (0 to 11)
ADL						
Pre-op	7	4	0	3	0	2.8(0 to 7)
Post-op	17	16	7	13	2	11.0 (0 to )*
ROM						
Pre-op	8	6	2	4	4	4.8 (2 to 8)
Post-op	24	28	4	28	12	19.2 (4 to 28)*
Strength						
Pre-op	0	0	0	0	0	0
Post-op	25	24	0	25	14	17.6 (0 to 25)
Total score						
Pre-op	15	13	3	10	4	9.0 (3 to 15)
Post-op	72	78	16	72	28	53.2 (16 to 78)*

* p<0.05 based on Wilcoxon Signed Ranks Test.

Abbreviations: ADL: activity of daily living, ROM: range of motion, pre-op: pre-operative, post-op: post-operative.

Range of motion, especially anterior elevation and abduction, also improved Table I. Four of the five patients were able to perform everyday activities such as bathing, combing hair without pain after their operation. Isometric strength in these four increased compared with their pre-operative assessment. At 90° abduction and wrist pronation, the mean power was 17.6 points (8kg) (range 0 to 25) (Table I) at the last follow-up. However, one patient showed no improvement in isometric strength as she could not elevate the arm to 90°.

**Fig. 2:**
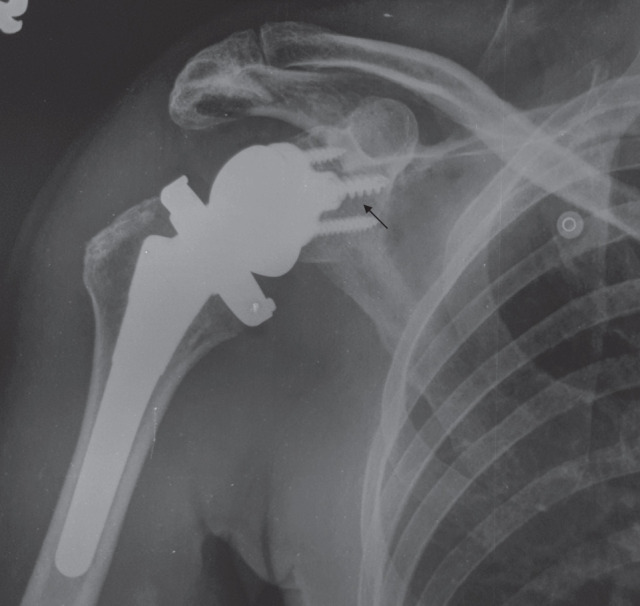
Anteroposterior radiographs showing a shoulder without radiographic signs of loosening after 10 months. No radiolucency is seen around the screws (arrow).

Although this was a short-term study, there were no early radiological complications such as implant migration, fractures or dislocations on post-operative radiographs at the last follow-up (Fig. 2).

## Discussion

The treatment for massive rotator cuff tears, with or without post-traumatic glenohumeral arthritis, remains a challenge. Reverse total shoulder arthroplasty (RTSA) has emerged as a reliable alternative for end glenohumeral shoulder pathology. Our study aims to evaluate the short-term functional and radiological outcomes following RTSA in a large public hospital in Malaysia.

Patients in our series presented with a significant functional deficit due to pain and loss of movement, having exhausted all forms of non-operative treatments. Their duration of illness ranged from six months to two years. The three most common diagnoses or indication of RTSA are fracture sequelae, rotator cuff arthropathy and osteoarthritis, with some patients having combined pathology^1,2,4,5^. Our findings are similar to other National Joint Replacement Registry reported internationally. Our patients’ demographics were also consistent with the epidemiological data from the USA, Finland, and Nordic population,^3,4,5^ with similar ages, and the dominant shoulder being more commonly affected^8^.

Many studies have shown promising functional outcomes following RTSA^7-10^. In 2005, Boileau *et al* revealed that RTSA led to an increase in Constant-Murley score from 17 to 58 points in 50 patients^11^. Young *et al* has evaluated early outcomes of RTSA in rheumatoid arthritis patients and showed that patients are satisfied with their result with remarkably better Constant-Murley score after a mean of 3.8 years^13^. Another study evaluating RTSA for massive rotator cuff tears in young patients after 5 to 15 years by Ek *et al* showed significant improvement in mean Constant score with minimal complications^14^.

From our small short-term study, both the Constant score and patient overall subjective outcomes showed significant improvement after undergoing RTSA, with similar results to other authors internationally. In our cohort, only one patient showed an unsatisfactory result. The reason for this poor outcome may be related to the cause of injury. She suffered post-traumatic osteoarthritis following a 4-part proximal humeral fracture six months before surgery. A study of RTSA outcomes according to aetiology by Wall *et al* suggested that patients with post-traumatic arthritis have a reduced functional outcome compared to other aetiologies, and our study supports this conclusion^12^. Furthermore, this patient was poorly compliant with her post-operative follow-up and physiotherapy sessions which might explain her poor outcome.

This present study has several limitations that must be considered when interpreting the results. Our research is a single-centre report; and some complication and outcomes may not be possible to discern. As retrospective administrative data sets were utilised, we could not compare RTSA and other treatments for the same aetiology. The limited number of patients in our cohort could have been inadequate data to achieve statistical significance in certain aspects. However, since RTSA is still a new procedure in Malaysia, our series of five patients treated with RTSA represents the first outcomes report in this country.

## Conclusions

This study has shown that reverse total shoulder arthroplasty can yield good short term outcomes when used to treat complex shoulder problems following proximal humeral fractures and cuff tear arthropathy in the Malaysian public healthcare setting. It should be considered a treatment for rotator cuff tears, severe arthritis and ≥ 3 parts proximal humeral fractures.
